# Association of selenium with type 2 diabetes and obesity: A univariate and multivariate Mendelian randomization study

**DOI:** 10.1097/MD.0000000000045338

**Published:** 2025-10-17

**Authors:** Lihui Cheng, Zheng Zeng, Hui Quan

**Affiliations:** a Clinical Laboratory, The Second Affiliated Hospital of Chengdu Medical College (National Nuclear Corporation 416 Hospital), Chengdu, China.

**Keywords:** body mass index, Mendelian randomization, selenium, type 2 diabetes

## Abstract

Selenium is associated with an increased risk of type 2 diabetes. However, whether the diabetogenic effect of selenium is mediated through increased body mass index (BMI) remains inconclusive. We conducted univariable and multivariable 2-sample Mendelian randomization (MR) to investigate the impact of selenium on the risk of type 2 diabetes and obesity. And further, we also tested whether the effect of selenium on diabetes was mediated through BMI. The inverse variance-weighted method was used as the main analysis to assess the effect estimates. Genetically proxied blood selenium (odds ratio [OR] 1.13, 95% confidence interval [CI] 1.10–1.16, *P* < .001; per standard deviation elevation of selenium level) and toenail and blood selenium (OR 1.13, 95% CI 1.06–1.19, *P* < .001) were both associated with increased risk of type 2 diabetes. In multivariable MR analysis, the direct effect of selenium on the risk of type 2 diabetes decreased (blood selenium: OR 1.07, 95% CI 1.04–1.10, *P* < .001; toenail and blood selenium: OR 1.09, 95% CI 1.05–1.14, *P* < .001). The diabetogenic effect of selenium was mediated through increased BMI by 25% to 50%. This MR study suggests that the genetically proxied selenium is associated with increased risk of diabetes and obesity. And the diabetogenic effect of selenium is partially mediated through increased BMI.

## 1. Introduction

Selenium is a trace mineral and is recognized as an essential element for human health. Selenoproteins are the major form of selenium and play a crucial role in a wide variety of physiological processes including antioxidant, anti-inflammatory, and production of active thyroid hormone.^[1]^ In consideration of these beneficial effects, selenium was considered as being a favorable therapy for disease control. Particularly, selenium supplementation was found to be effective and safe in treating Hashimoto thyroiditis in a systematic review and meta-analysis.^[2]^ Furthermore, selenium supplementation has been introduced into food products in many countries.^[3,4]^ However, selenium supplementation was not found to be beneficial for many chronic diseases.^[5–7]^

The relationship between selenium and type 2 diabetes is complex. Low selenium exposure was suggested to be associated with type 2 diabetes because of the assumption that an element deemed to be “antioxidant” should be useful in reducing the risk of disease associated with oxidative stress. However, a surprising positive association between selenium supplementation and diabetes was revealed in 2007 by Stranges et al.^[8]^ Several meta-analysis studies of observational and randomized controlled studies all suggested that selenium supplementation was associated with increased risk of type 2 diabetes.^[9–11]^ Therefore, the role of selenium and diabetes mellitus is controversial.^[12,13]^

Unlike observational and randomized controlled studies, Mendelian randomization (MR) uses genetic variation to address causal effect questions.^[14]^ This could avoid unobserved confounding of the exposure in observational studies. Because the genetic variation is generally randomly allocated at conception, MR provide an approach to evaluate causal questions through mimicking randomized controlled trial.^[15]^ In a previous MR study by Rath et al, genetically predicted selenium was associated with increased risk of type 2 diabetes (odds ratio [OR] 1.27, 95% confidence interval [CI] 1.07–1.50, *P* = .006).^[16]^

Obesity plays a critical role in the development of type 2 diabetes.^[17]^ Whether obesity mediate the diabetogenic effects of selenium supplementation is inconclusive. Several previous studies found that selenium concentrations are higher in obese individuals.^[18,19]^ However, inconsistent results were revealed. A systematic review and meta-analysis did not find significant association between overweight/obesity and plasma/serum selenium level, although overweight/obese individuals had a trend of higher levels of selenium in the hair.^[20]^ Furthermore, Serum selenium was found to accelerate the development of metabolic disorders in a metabolically healthy obese.^[21]^ A case-control study by Lu et al demonstrated that the association of selenium level with diabetes were largely decreased after adjusting for waist circumference and homeostatic model assessment-insulin resistance.^[22]^

Mediation analysis is a field of analysis that investigate the causal pathways or mechanism by which 1 exposure influences an outcome. Multivariable MR analysis is a recent extension to MR that can be used to estimate the mediation effects.^[23]^ In this study, we used MR study to assess the impact of selenium on diabetes and obesity, and further to test the hypothesis that diabetogenic effect of selenium is mediated partly through increased body mass index (BMI).

## 2. Materials and methods

We followed the guidelines for performing MR investigations^[24]^ and STROBE-MR^[25]^ to perform and report the results of the MR analysis. The present study is based on publicly available summary-level data from genome-wide association study (GWAS). All of these studies have received approval from the respective institutional review boards. Detailed information regarding the GWAS studies utilized in this study is provided in Table 1. To investigate the causal relationship between selenium and diabetes as well as obesity, we performed MR analysis of selenium level with diabetes and BMI, respectively. Three core assumptions of MR analysis were met. The approach to mediation analysis was depicted in Figure S1, Supplemental Digital Content, https://links.lww.com/MD/Q413.

**Table 1:** Summary information of the GWAS studies used in this study.

Trait	Source	Ancestry	Sample size or case/control	Author and year	Use in this study
Blood selenium	Australian twins and their families and pregnant women from UK	European	5477	Evans 2013	Exposure
T&B selenium	A meta-analysis proxying toenail and blood selenium	European	9739	Corneils 2015	Exposure
Type 2 diabetes	DIAMANTE	European	80,154/8,53,816	Mahajan 2022	Outcome
BMI	GIANT consortium and UK biobank	European	8,06,834	Pulit 2019	Outcome

BMI = body mass index, DIAMANTE = diabetes meta-analysis of trans-ethnic, GIANT = genetic investigation of anthropometric traits, GWAS = genome-wide association study, T&B = toenail and blood.

### 2.1. Selection of genetic variables

Single nucleotide polymorphisms (SNPs) strongly associated (*P* < 5 × 10^−8^) with blood selenium level from 5477 European ancestry (2603 Australian twins and their families and 2875 pregnant women from UK) were obtained.^[26]^ Linkage disequilibrium was pruned (*r*^2^ < 0.3). We used PhenoScanner v2 to assess the possibility of horizontal pleiotropy among the instrumental variables.

Plasma and toenail selenium concentrations were significantly correlated, while Toenail selenium may reflect a longer average duration of exposure. Both blood and toenail selenium similarly reflect selenium exposure and are used as markers of selenium exposure in epidemiologic studies. Therefore, the SNPs from a genome-wide meta-analysis proxying toenail and blood selenium level (T&B) were also used. Toenail selenium level were obtained from 4162 participants in the United States.^[27]^

### 2.2. Outcome data sources

The diabetes meta-analysis of trans-ethnic association studies consortium assembled the most ancestrally diverse collection of GWAS of diabetes including 1,80,834 cases and 11,59,055 controls.^[28]^ In this study, we used the meta-analysis of type 2 diabetes genetic associations in 80,154 cases and 8,53,816 controls of European ancestry as the outcome.

### 2.3. Genetic associations with BMI

We obtained genetic associations with BMI from a meta-analysis of the genetic investigation of anthropometric traits consortium and UK biobank (sample size 8,06,834).^[29]^

The genetic investigation of anthropometric traits consortium is a collaboration that seeks to identify genetic loci that modulate human body size and shape including height and obesity. The UK biobank is a large-scale biomedical database which is investigating the genetic predisposition and environmental exposure to the development of disease from half a million UK participants. There were no individuals that overlap between the exposure and outcome studies.

### 2.4. Statistical analysis

Two sample MR method was used in this study to assess the effect of genetically predicted blood selenium level and T&B selenium level on type 2 diabetes (univariable MR analysis). The inverse variance-weighted method with multiplicative random effects was used as the primary analysis method. The MR-Egger, median-based methods, and MR-PRESSO would be used to perform sensitivity analysis to assess the robustness. Leave-one-out analysis was also used to assess the sensitivity.

Multivariable MR analysis by using inverse variance-weighted method was utilized to assess the associations of genetically proxied selenium with type 2 diabetes (direct effect). The total effect is defined as the net effect of genetically proxied selenium on type 2 diabetes (the univariable MR estimate). The indirect effect is defined as the effect of genetically proxied selenium on type 2 diabetes mediated through BMI (the univariable MR estimate minus the multivariable MR estimate). The proportion of mediation was calculated by dividing the indirect effect into the total effect (Fig. S1, Supplemental Digital Content, https://links.lww.com/MD/Q413).

Statistical analysis was conducted in R (version 4.2.0). The “TwoSampleMR” and “MendelianRandomization” package were used to perform the univariable and multivariable MR analysis. The “MR-PRESSO” package was used to estimate the horizontal pleiotropy. The “forestplot” package was used to visualize the results. Bonferroni correction was used to account for multiple testing, and associations with *P* value ≤ .01 (.05/3 = .017) are described as significant. *P* value between .01 and .05 was considered nominally significant. The protocol was not preregistered.

## 3. Results

A total of 22 SNPs were used as the instrumental variable for blood selenium level. Of 12 SNPs identified for T&B selenium, one (rs558133) was not available in diabetes meta-analysis of trans-ethnic, and thus 11 SNPs were selected to proxy the T&B selenium (Tables S1 and S2, Supplemental Digital Content, https://links.lww.com/MD/Q414). The SNPs proxying blood and T&B selenium level explained ~4.5%, ~4% of variance in selenium, respectively.^[16]^ The corresponding *F*-statistic ranges for these instruments were 31.75 to 119.15 and 30.51 to 174.21, respectively, suggesting that the instruments were unlikely to suffer from weak instrument bias.

**Figure 1.**
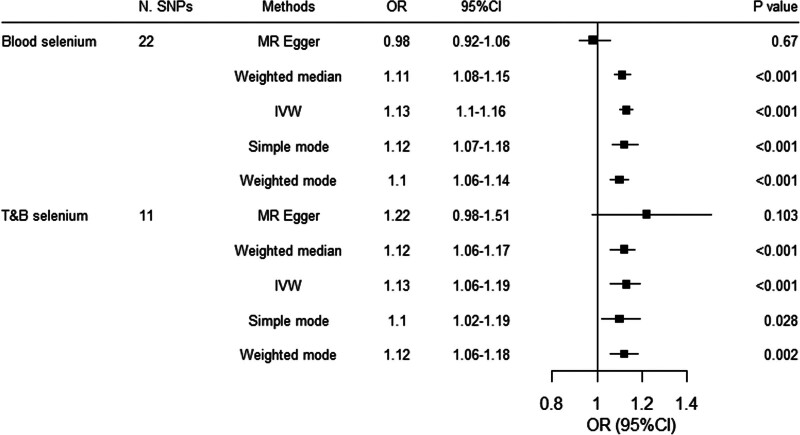
Associations between genetically proxied selenium and the risk of type 2 diaebtes. CI = confidence interval, IVW = inverse variance-weighted, MR = Mendelian randomization, OR = odds ratio, SNPs = single nucleotide polymorphisms.

Genetically proxied blood selenium (OR 1.13, 95% CI 1.10–1.16, *P* < .001; per standard deviation [SD] elevation of selenium level) and T&B selenium (OR 1.13, 95% CI 1.06–1.19, *P* < .001) were both associated with increased risk of type 2 diabetes (Fig. 1). The associations remained consistent in the weighted median, the simple mode, and the weighted mode methods, while the association was not significant in the MR-Egger method for both blood and T&B selenium. The Cochran’s *Q* test did not detect any evidence of heterogeneity (*P* = .082 and .431, respectively). No horizontal pleiotropy was observed based on MR-Egger intercept (*P* = .145 and .466, respectively). No outliers were identified with MR-PRESSSO.

**Figure 2.**
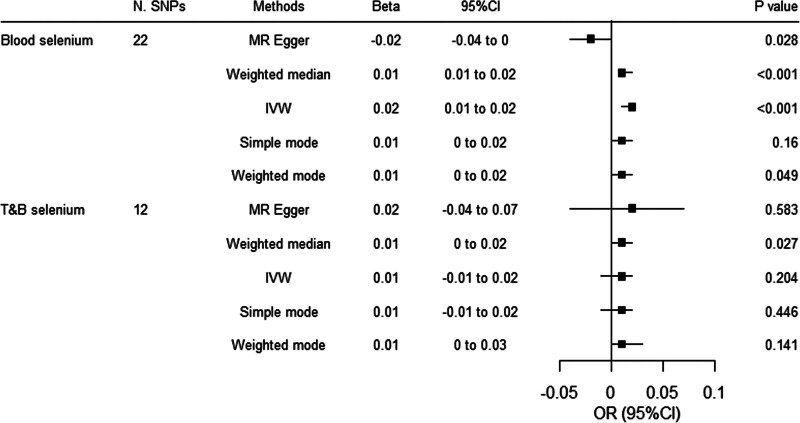
Associations between genetically proxied selenium and risk of body mass index. CI = confidence interval, IVW = inverse variance-weighted, MR = Mendelian randomization, OR = odds ratio, SNPs = single nucleotide polymorphisms .

To estimate the effect of selenium on type 2 diabetes mediated through BMI, we first evaluated the association of genetically proxied selenium with BMI and found that per SD increase of genetically proxied blood selenium increases BMI by 0.02 SD units (95% CI 0.01–0.02, *P* < .001). However, the effect was not observed when T&B selenium was used as instrumental variables (Fig. 2). No outliers were identified with MR-PRESSSO.

**Figure 3.**
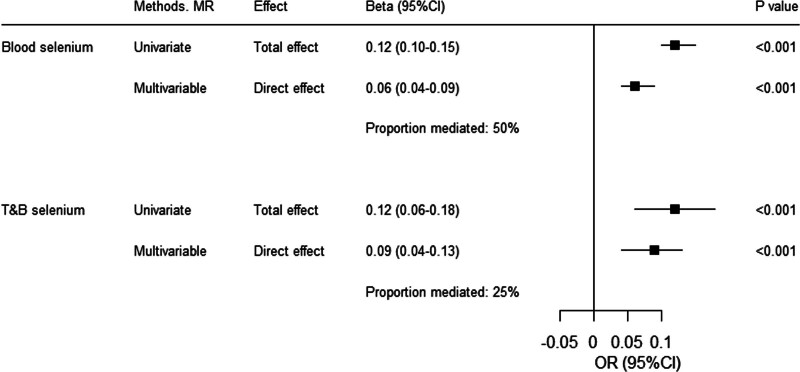
Multivariable analysis of genetically proxied selenium and type 2 diabetes. CI = confidence interval, IVW = inverse variance-weighted, MR = Mendelian randomization, OR = odds ratio.

In multivariable MR analysis, we observed less evidence of a direct effect of genetically proxied selenium on the risk of type 2 diabetes (OR 1.07, 95% CI 1.04–1.10, *P* < .001). The calculated proportion mediated through increased BMI was 50%. When T&B selenium was used as instrumental variables, Similar findings were observed (OR 1.09, 95% CI 1.05–1.14, *P* < .001). The diabetogenic effect of genetically proxied T&B selenium was mediated through increased BMI by 25% (Fig. 3).

## 4. Discussion

In this MR study, we found that selenium was associated with increased risk of type 2 diabetes. The diabetogenic effect of selenium was partially mediated through increased BMI.

The finding that high level of selenium would increase the risk of type 2 diabetes was consistent with previous observational and clinical trials.^[9–11]^ We also formally investigated whether increased BMI mediated the diabetogenic effect of selenium. Observational studies revealed that selenium was associated with obesity.^[18,19,30]^ A previous meta-analysis of nutritional status of selenium in overweight and obesity demonstrated that there was no significant difference between overweight/obesity and eutrophic groups in terms of dietary intake and plasma/serum levels of selenium.^[20]^ In animal experiments, selenium supplementation was unable to prevent obesity.^[31]^ For the first time, we demonstrated that genetically proxied blood selenium was associated with increased risk of obesity by utilizing MR analysis. Furthermore, BMI mediated diabetogenic effect of selenium was first revealed in this study. However, it is worth noting that the association between selenium and BMI was not observed when T&B selenium was used as instrumental variables.

It is well known that obesity and insulin resistance are closely related. Previous observational studies revealed that selenium was associated with insulin resistance.^[32]^ In animal models, it is also found that a high-selenium diet induces insulin resistance in gestating rats and their offspring.^[33]^ The detrimental effect of selenium on insulin sensitivity may contribute to dysfunction of target organs such as the pancreas, liver, kidney and adipose tissue.^[34]^ Association between selenium and insulin resistance indirectly support the results of this study.

The results of this study further improved our understanding of pathophysiological processes leading to diabetes by selenium. The results have implications for clinical practice. First, the deleterious effect of selenium on obesity and diabetes questions the selenium supplementation in the general population. Second, as an important trace nutrient, selenium deficiency has been associated with an increased risk of mortality, poor immune function, and cognitive decline.^[1]^ Selenium supplementation might still be a reasonable therapeutic measure in participants with selenium deficiency. In clinical practice, it is critical to realize that the absence of problem to be solved – selenium deficiency should be an important prerequisite for a nutrient intervention trial.^[35]^ Therefore, selenium supplementation should be reserved for individuals with the problem of selenium deficiency.

There are several strengths. First, the MR design could minimize bias from residual confounding and reverse association. Second, both the SNPs of selenium from blood and toenail were utilized as instrumental variable. The study also has limitations. First, the selenium level that used to build up the association of selenium and SNPs was assessed at a single timepoint. It is unclear whether the selenium exposure vary over time. Second, the association of selenium status and disease risk might be U-shaped but rather linear. However, the MR assessed the association based on a linear relationship. Third, although MR analysis can minimize bias pleiotropy and reverse causal inference, the possibility of residual pleiotropy and reverse causality may also bias the estimates of the MR analysis. Fourth, the association between T&B selenium and BMI was not observed. Therefore, further research should be conducted to clarify the findings of this study.

In conclusion, we found that genetically predicted selenium was associated with increased risk of diabetes and obesity. The diabetogenic effect of selenium was partially mediated through increased BMI.

## Acknowledgments

We want to acknowledge all the participants and investigators of DIAMANTE consortium, GIANT consortium and UK biobank project.

## Author contributions

**Conceptualization:** Lihui Cheng, Zheng Zeng, Hui Quan.

**Formal analysis:** Lihui Cheng.

**Methodology:** Lihui Cheng, Zheng Zeng.

**Software:** Zheng Zeng.

**Supervision:** Hui Quan.

**Writing** – **original draft:** Lihui Cheng.

**Writing** – **review & editing:** Hui Quan.

## Supplementary Material




