# Supplementary material for: Association of selenium with type 2 diabetes and obesity: A univariate and multivariate Mendelian randomization study
Source: Medicine (Baltimore). 2025 Oct 17;104(42):e45338. doi: 10.1097/MD.0000000000045338 (PMC12537198; doi:10.1097/MD.0000000000045338)
Supplement: Supplementary file 1 [file medi-104-e45338-s001.docx]

**Supplementary Figure 1**. Diagram of mendelian randomization and mediation analysis.


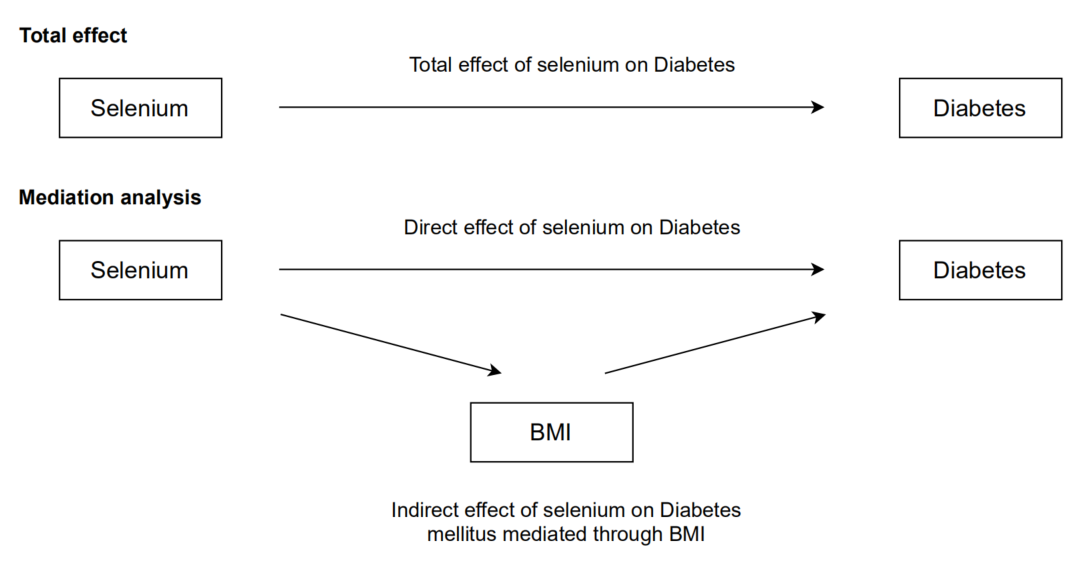


The total effect is defined as the net effect of genetically proxied selenium on type 2 diabetes (the univariable MR estimate). The indirect effect is defined as the effect of genetically proxied selenium on type 2 diabetes mediated through BMI (the univariable MR estimate minus the multivariable MR estimate). The proportion of mediation was calculated by dividing the indirect effect into the total effect.
