# Supplementary material for: Association of selenium with type 2 diabetes and obesity: A univariate and multivariate Mendelian randomization study
Source: Medicine (Baltimore). 2025 Oct 17;104(42):e45338. doi: 10.1097/MD.0000000000045338 (PMC12537198; doi:10.1097/MD.0000000000045338)
Supplement: Supplementary file 2 [file medi-104-e45338-s002.docx]

**Supplementary Table** 1. Genetic variables of blood selenium used in the Mendelian randomization study

| SNP | Chr | position | Effect allele | Ref allele | EAF | Beta | SE | P value | F statistics | Gene |
| --- | --- | --- | --- | --- | --- | --- | --- | --- | --- | --- |
| rs163124 | 5 | 78283003 | G | T | 0.28 | 0.1483 | 0.034 | 5.16E-12 | 47.58 | ARSB |
| rs163132 | 5 | 78285921 | C | T | 0.23 | 0.1683 | 0.035 | 7.32E-14 | 55.95 | ARSB |
| rs672413 | 5 | 78278229 | A | G | 0.32 | 0.1172 | 0.033 | 1.68E-08 | 31.75 | ARSB |
| rs7700970 | 5 | 78411324 | T | C | 0.32 | 0.2124 | 0.037 | 1.72E-18 | 77.03 | BHMT |
| rs9293761 | 5 | 78290215 | G | A | 0.56 | 0.1857 | 0.032 | 1.24E-18 | 77.47 | DMGDH |
| rs8180502 | 5 | 78477017 | G | A | 0.7 | 0.1205 | 0.035 | 4.70E-08 | 29.73 | DMGDH |
| rs586199 | 5 | 78397980 | G | A | 0.5 | 0.2011 | 0.029 | 2.37E-26 | 113.21 | DMGDH |
| rs705415 | 5 | 78291960 | C | T | 0.88 | 0.2321 | 0.059 | 4.56E-10 | 38.93 | DMGDH |
| rs2445887 | 5 | 78310044 | A | G | 0.46 | 0.1606 | 0.031 | 7.63E-16 | 65.13 | DMHDH |
| rs949644 | 5 | 78442351 | A | G | 0.67 | 0.1674 | 0.031 | 2.03E-16 | 67.34 | DMGDH |
| rs478651 | 5 | 78290682 | T | C | 0.48 | 0.1624 | 0.035 | 3.53E-13 | 53.03 | DMGDH |
| rs7710824 | 5 | 78297271 | A | C | 0.28 | 0.1612 | 0.035 | 3.86E-13 | 52.73 | DMHDH |
| rs921943 | 5 | 78316476 | T | C | 0.3 | 0.2456 | 0.034 | 9.40E-28 | 119.15 | DMGDH |
| rs17823744 | 5 | 78344976 | G | A | 0.13 | 0.2852 | 0.045 | 8.93E-22 | 92.21 | DMGDH |
| rs16876498 | 5 | 78402594 | C | T | 0.1 | 0.3079 | 0.048 | 1.23E-22 | 96.15 | DMGDH |
| rs1915706 | 5 | 78436211 | C | T | 0.62 | 0.1606 | 0.031 | 3.04E-15 | 61.98 | DMGDH |
| rs3797535 | 5 | 78300397 | T | C | 0.1 | 0.2132 | 0.057 | 2.42E-09 | 35.66 | DMGDH |
| rs248380 | 5 | 78331741 | T | C | 0.51 | 0.2056 | 0.029 | 1.84E-27 | 118.34 | DMGDH |
| rs10514151 | 5 | 78303487 | T | C | 0.06 | 0.2091 | 0.059 | 1.42E-08 | 32.11 | DMGDH |
| rs16876394 | 5 | 78346769 | C | T | 0.1 | 0.3047 | 0.048 | 3.32E-22 | 94.16 | DMGDH |
| rs10514159 | 5 | 78596044 | C | T | 0.62 | 0.1346 | 0.031 | 7.48E-12 | 47.16 | JMY |
| rs9293769 | 5 | 78629346 | T | C | 0.6 | 0.1229 | 0.032 | 1.75E-09 | 36.29 | JMY |

SNP, single nucleotide polymorphism; Chr, chromosome; Ref allele, reference allele; EAF, effect allele frequency.

**Supplementary Table 2**. Genetic variables of toenail/blood selenium used in the Mendelian randomization study

| SNP | Chr | position | Effect allele | Ref allele | EAF | Beta | SE | P value | F statistics | Gene |
| --- | --- | --- | --- | --- | --- | --- | --- | --- | --- | --- |
| rs6586282 | 21 | 44478497 | T | C | 0.17 | -0.11273 | 0.019139 | 3.96E-09 | 34.71 | CBSL |
| rs1789953 | 21 | 44482936 | T | C | 0.14 | 0.114396 | 0.020724 | 3.40E-08 | 30.51 | CBSL |
| rs234709 | 21 | 44486964 | T | C | 0.45 | -0.0844 | 0.014452 | 5.23E-09 | 34.16 | CBSL |
| rs672413 | 5 | 78278229 | A | G | 0.32 | 0.115921 | 0.015395 | 5.21E-14 | 56.81 | ARSB |
| rs705415 | 5 | 78291960 | T | C | 0.14 | -0.14113 | 0.022653 | 4.64E-10 | 38.90 | DMGDH |
| rs3797535 | 5 | 78300397 | T | C | 0.08 | 0.210104 | 0.026462 | 2.05E-15 | 63.22 | DMGDH |
| rs11951068 | 5 | 78304314 | A | G | 0.07 | 0.189249 | 0.028162 | 1.86E-11 | 45.22 | DMGDH |
| rs921943 | 5 | 78316476 | T | C | 0.29 | 0.206719 | 0.015732 | 1.90E-39 | 174.21 | DMGDH |
| rs10944 | 5 | 78385845 | T | G | 0.49 | 0.180759 | 0.014289 | 1.13E-36 | 161.30 | BHMT2 |
| rs567754 | 5 | 78416416 | T | C | 0.34 | -0.13792 | 0.015139 | 8.38E-20 | 83.31 | BHMT |
| rs6859667 | 5 | 78745042 | T | C | 0.96 | -0.25371 | 0.036663 | 4.40E-12 | 48.06 | HOMER1 |

SNP, single nucleotide polymorphism; Chr, chromosome; Ref allele, reference allele; EAF, effect allele frequency.
